# Rapid Point-of-Care Assessment of Paracetamol Intoxication via an Integrated Electrochemical–Colorimetric Method

**DOI:** 10.1021/acsomega.5c12147

**Published:** 2026-02-24

**Authors:** Anne A. Macedo, Dilton M. Pimentel, Karla A. O. Souza, José L. Costa, Cláudia M. Rocha, Clésia C. Nascentes, Ângelo de Fátima, Luciano C. Arantes, Fateme Ebrahimi, Bertold Rasche, Wallans T. P. dos Santos

**Affiliations:** † Departaments of Chemistry and Pharmacy, 74380 Universidade Federal Dos Vales Do Jequitinhonha e Mucuri (UFVJM), 39100000 Diamantina, Minas Gerais, Brazil; ‡ Laboratório Integrado de Pesquisas Do Vale Do Jequitinhonha, PRPPG-UFVJM, 39100000 Diamantina, Minas Gerais, Brazil; § Departament of Chemistry, Instituto de Ciências Exatas, 28114 Universidade Federal de Minas Gerais (UFMG), 31270-901 Belo Horizonte, Minas Gerais, Brazil; ∥ Instituto Nacional de Ciência e Tecnologia Sobre Substâncias Psicoativas (INCT-SP), UFMG, 31270-901 Belo Horizonte, Minas Gerais, Brazil; ⊥ Faculdade de Ciências Médicas, 28132 Universidade Estadual de Campinas (UNICAMP),13083-859 Campinas,São Paulo, Brazil; # Centro de Informação e Assistência Toxicológica de Campinas (CIATox-Campinas), UNICAMP, 13083-859 Campinas, São Paulo, Brazil; ¶ Faculdade de Ciências Farmacêuticas, UNICAMP, 13083-859 Campinas, São Paulo, Brazil; ∇ Institute of Inorganic Chemistry, 9149 University of Stuttgart, 70569 Stuttgart, Germany

## Abstract

This work addresses the detection and quantification of paracetamol (PAR) in human serum using a point-of-care approach, an essential assessment for intoxication cases in clinical analysis. Electrochemical and colorimetric methods have been widely reported for simple and fast determination of PAR in biological samples. However, most of these previous portable methods have neglected some aspects for a real-world clinical application of PAR intoxication, such as its toxic concentration range and a proper interference study in biological matrices. To fill this gap, we introduce a novel integrated sensing approach, combining colorimetric and electrochemical techniques for determining PAR. In contrast to other sensors, this work employs a simple screen-printed graphite electrode (SPE-Gr) combined with a colorimetric reagent (CR) for the rapid and selective detection of PAR in human serum. Analysis occurs via dual confirmation: after reaction between PAR and CR, first a color change can indicate intoxication levels, and the generated product is subsequently accurately quantified by square-wave adsorptive stripping voltammetry using a SPE-Gr. A wide linear range (4.5 to 1000 mg L^–1^) for determining PAR was obtained, with a limit of detection (1.2 mg L^–1^), covering its therapeutic and toxic concentrations for assessment of intoxication cases. High reproducibility was demonstrated by low relative standard deviations (RSDs < 2.0%) and consistent responses obtained with different disposable SPEs-Gr (*n* = 3). Analysis in real human serum with a comprehensive interference study showed a high selectivity and reliability of the proposed method for PAR detection in biological samples. Therefore, our combined electrochemical-colorimetric method provides a suitable point-of-care sensor for PAR determination, enabling a prompt and straightforward diagnosis to reduce mortality in intoxication cases.

## Graphical abstract

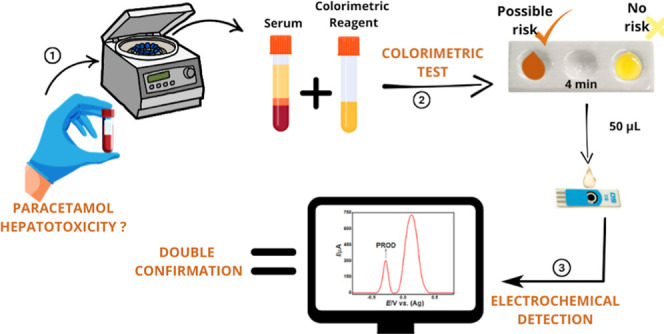

## Introduction

Paracetamol (PAR), also known as acetaminophen, is one of the most widely used analgesics and antipyretics due to its effectiveness and over-the-counter availability.^1^ Despite its popularity and inclusion on the world health organization (WHO) list of essential medicines,^2^ PAR has a relatively narrow therapeutic range: its toxic dose is only about 10 times the therapeutic dose, making it a leading cause of overdose, both accidental and intentional, and the second leading cause of liver transplants globally.^3–6^ In the United States and the United Kingdom, for example, PAR toxicity results in more than 50,000 hospitalizations annually, including serious and fatal cases.^3–7^ PAR is primarily metabolized in the liver, with over 90% processed through glucuronic acid conjugation, sulfation, and oxidation. Oxidation produces *N*-acetyl-*p*-benzoquinone imine, a reactive metabolite neutralized by glutathione (GSH). However, in acute overdoses—starting at 10 g or 200 mg/kg within 24 h—GSH reserves are depleted, resulting in severe hepatotoxicity.^5,8^ To assess the risk of hepatotoxicity in cases of acute PAR ingestion, the Rumack–Matthew nomogram is used.^9^ This tool correlates serum PAR concentration with the time elapsed since ingestion and is most effective within the first 24 h, particularly between 4- and 8 h postexposure, a critical window for administering the antidote, *N*-acetylcysteine. This compound replenishes GSH levels by being hydrolyzed into cysteine, mitigating liver damage and preventing its failure.^5,9,10^ Consequently, serum PAR concentration measures are critical for confirming the diagnosis of acute intoxication and guiding antidote administration.^11^

Despite the widespread use of PAR, the clinical management of acute intoxication still faces significant challenges, particularly with regard to timely and equitable access to reliable diagnostic tools. In many healthcare settings, especially those with limited infrastructure, rapid determination of serum PAR concentrations is constrained by cost, limited equipment availability, and operational complexity. These limitations can compromise clinical decision-making within the critical therapeutic window and constitute a barrier to global health goals aimed at reducing preventable morbidity and mortality.^13^

Screening and testing for PAR within the first 24 h after ingestion are critical, as patients with toxic doses often remain asymptomatic during this period. In this context, the availability of rapid and accurate analytical methods to quantify the serum PAR levels is crucial in hospital settings, as they allow the assessment and interpretation of toxicity levels based on the Rumack–Matthew nomogram, aiding in rapid and effective clinical decision-making.^9,12^ PAR detection in these settings typically involves the use of immunoassays, such as ELISA, which are the most common choice due to their rapid screening and specificity but depend on reagent availability and laboratory infrastructure.^13^ Spectrophotometric techniques are also used, mainly in hospitals with limited resources,^13^ due to their simplicity and cost-effectiveness, although their lower selectivity and greater susceptibility to interference make them less ideal for emergency results. Gas or liquid chromatographic techniques, particularly those coupled with mass spectrometry, such as GC–MS and LC–MS, are highly precise for the confirmation of drugs in clinical analysis. However, these chromatographic methods are costly and time-consuming, making them unfavorable as a rapid test in cases of intoxication and therefore in emergency settings.^13,14^ In clinical toxicology, serum/plasma remains the matrix of choice for confirming PAR poisoning because it directly reflects the pharmacologically active fraction of the drug and serves as the reference matrix for the Rumack–Matthew nomogram.^9^ However, its complex biochemical composition, rich in electroactive compounds such as uric acid and ascorbic acid, poses analytical challenges and underscores the need for selective and interference-resistant detection strategies.

The electrochemical methods have been widely reported for detecting PAR in biological samples,^11^ providing a fast and selective screening test, with high sensitivity and low cost for application in clinical analysis. However, most of the reported methods are based on modified electrodes, which present some practical limitations for clinical settings.^11^ To increase the portability and simplicity for applications in on-site analysis, the electrochemical sensors based on screen-printed electrodes (SPEs) have been successfully applied for detecting many drugs in different samples,^15–17^ including for determining PAR.^18–20^ SPEs are commonly used as disposable sensors, which can reduce the risk of contamination between samples in a clinical analysis. Furthermore, the use of SPEs is widely available commercially, facilitating the standardization and replacement in clinical laboratories. Among these SPEs, the working electrodes based on graphite (SPE-Gr) are the simplest and low-cost but have not yet been applied for PAR detection in human serum samples.

Voltammetry is the main electroanalytical technique that has been used for detecting PAR in biological samples,^11^ notably using square wave (SWV) and differential pulse excitation (DPV). The adsorptive stripping voltammetry (AdSV) technique can increase sensitivity in electroanalysis when the electroactive molecule demonstrates an adsorption process on the working electrode surface.^21,22^ Even though PAR has demonstrated this behavior with different working electrodes, the AdSV technique has been little explored for its detection in biological samples,^11^ with only some works using square wave adsorptive stripping voltammetry (SWAdSV)^23^ and differential pulse adsorptive stripping voltammetry (DPAdSV).^24,25^

Although these electroanalytical methods have been successfully applied for PAR detection, a recent critical review,^11^ with more than 250 published articles, showed that the vast majority of electrochemical sensors remain unadopted or unexplored for a real clinical application in intoxication cases.^11^ Most of these previous reports have also been neglected a comprehensive interference study, particularly considering physiologically relevant concentrations of electroactive compounds such as ascorbic acid (AA) and uric acid (UA), which is crucial for real-world clinical applications.^11^ Given these practical and clinical constraints, colorimetric sensing strategies are particularly attractive because they combine operational simplicity with a rapid visual readout and have been successfully integrated into diverse analytical platforms, with applications ranging from clinical diagnostics to environmental analysis, and biological monitoring.^26^ Colorimetric methods for PAR detection have also been described in the literature, including applications in human serum/plasma,^27–29^ but typically require complex sample pretreatment or advanced nanomaterials, limiting their practicality for rapid toxicological screening. Importantly, Emerson’s reagent has not yet been applied to PAR detection, despite its strong reactivity toward phenolic groups^30^

To address these limitations and advance the field of chemical detection for clinical applications, combined methods using colorimetric and voltammetric techniques have emerged as a promising alternative for PAR detection. Some recent applications have been reported using this approach for the highly selective identification of drugs in forensic samples.^31–33^ To the best of our knowledge, only two studies have combined electrochemical and colorimetric techniques to detect PAR,^34,35^ where both using modified electrodes with nanomaterials. Although these methods demonstrated good analytical performance, their modified sensors can limit their scalability and application as a point-of-care test. Moreover, the combined use of electrochemical and colorimetric techniques remains underexplored for the clinical diagnosis of PAR in intoxication cases, despite the clear need for low-cost, rapid, and simple tests suitable for routine analyses. Accordingly, integrating a straightforward colorimetric reaction with an electrochemical readout provides a practical route to enhance the selectivity and robustness in complex serum matrices while maintaining the speed and operational simplicity required for point-of-care use.

In light of the above-mentioned insights, this work introduces a novel integrated sensing method that combines colorimetric and voltammetric assays for determining PAR in human serum samples. The proposed method offers a rapid and straightforward colorimetric assay based on Emerson’s reagent, integrated with simple and sensitive electrochemical detection employing commercial SPE-Gr and the SWAdSV technique. The combined colorimetric-electrochemical detection method can selectively detect and quantify PAR in clinical analyses, providing dual identification for the presence of this drug in complex serum samples. The colorimetric assay detects the presence of PAR by color change (1), and the electrochemical assay (2) can quantify PAR through a specific signal from the colorimetric reaction product. Furthermore, we present, for the first time, a comprehensive interference study and demonstrate adequate analytical performance for potential applications in the clinical diagnosis of PAR intoxication. Accordingly, this work offers high portability, operational simplicity, and improved selectivity for monitoring PAR intoxication cases, representing a conceptual advancement in sensor development for practical diagnostic applications.

## Experimental Section

### Chemicals and Samples

All solutions were prepared with deionized water with a resistivity of not less than 18.2 MΩ.cm^–1^ (at 25 °C) obtained by using the Milli-Q system (Millipore, USA). The reagents 4-aminoantipyrine (ACS Científica), potassium ferricyanide (Neon), sodium phosphate (ACS Científica), and boric acid (ACS Científica) were used for the colorimetric test. The analytical standard of PAR (Sigma Chemical Co) was diluted in deionized water for the subsequent analyses. A serum sample was collected from a patient with the suspicion of PAR intoxication at Campinas Poison Control Center (CAAE: 75425223.4.0000.5404).

### Instrumental and Apparatus

All voltammetric experiments were carried out using a μPGSTAT 101 N potentiostat (Metrohm Autolab BV, Utrecht, the Netherlands) controlled by NOVA 2.1 software. The electrochemical behavior of all compounds was studied using commercial SPEs from Metrohm DropSens (Oviedo, Spain), with a 4 mm diameter graphite working electrode (SPE-Gr, model DRP-110), a carbon auxiliary electrode, and a silver pseudoreference electrode.

### Colorimetric Procedure

100 μL of PAR or serum sample, at different concentrations, was added to 10 μL of 4-aminoantipyrine (4-AAP) (2% m/v), 30 μL of potassium ferricyanide (K_3_Fe­(CN)_6_) (8% m/v), and 100 μL of buffer, 0.1 mol L^–1^ (phosphate for pHs 2, 3, 6.2, 7, 8, 11, and 12; acetate for pHs 4 and 5; and borate for pHs 9 and 10).^30^ This reaction was adapted from the original colorimetric reagent used for phenolic group detection, well-known as Emerson’s reagent.^30^ All reagent stock solutions were homogenized prior to use, and no precipitation or persistent turbidity was observed after mixing the colorimetric reagent with the buffer solutions over the investigated pH range (2–12).

### Electrochemical Measurements

The electrochemical analyses were performed with and without colorimetric reagents. Five successive scans, by cyclic voltammetry (CV), in the potential window of −1.0 V to +1.0 V (vs. Ag) at a scan rate of 100 mV s^–1^, were performed with conditioning of SPE-Gr before each measurement. Electrochemical studies were performed by CV at SPE-Gr, using different scan rates and different pH values (2 a 12) to optimize the system. The voltammetric detection of PAR was optimized using the SWAdSV technique. Optimization involved varying several parameters: accumulation time in the range of 0 to 8 min, pulse amplitude between 10 and 100 mV, step potential from 1 to 10 mV, and frequency from 10 to 100 Hz. All electrochemical measurements were performed in triplicate. The theorical limits of detection (LOD) and quantification were calculated according to the guidelines of the International Union of Pure and Applied Chemistry (IUPAC) guidelines.^36^ The LOD was defined as LOD = 3 sb/S, and the LOQ was defined as LOQ = 10 sb/S, where sb represents the standard deviation of ten consecutive blank measurements (*n* = 10) using the supporting electrolyte, and *S* is the slope (sensitivity) of the calibration curve. The voltammograms obtained by SWAdSV were processed with background subtraction by using GPES 4.9 software. Method repeatability was assessed by performing three consecutive measurements under identical conditions using the same SPE-Gr, while reproducibility was evaluated using different disposable SPE-Gr electrodes (*n* = 3).

### General Procedure for Application to Human Serum Samples

The colorimetric test was performed in triplicate using colorimetric reagent (CR), prepared with volumes and concentrations mentioned in the colorimetric procedure item, as a blank solution. Human blood samples were centrifuged at 4000 rpm for 10 min to separate the serum. Subsequently, 100 μL of serum sample was added to a spot plate, followed by the sequential addition of 10 μL of 4-AAP (2% m/v), 30 μL of K_3_ [Fe (CN)_6_] (8% m/v), and 100 μL of phosphate buffer (PB) 0.1 mol L^–1^, at pH 12.0. The reaction was allowed to proceed for 3 min.

Subsequently, 50 μL of this solution was applied to the electrode, and scanning was performed with an accumulation time of 1.0 min, a pulse amplitude of 100 mV, a step potential of 3 mV, and a frequency of 60 Hz.

## Results and Discussion

### Colorimetric Method

The colorimetric test adopted was based on the Emerson Reaction,^30^ in which PAR reacts with CR in a suitable buffer. Optimal pH for this colorimetric reaction was studied by varying the pH between 2 and 12. Different buffer solutions (phosphate for pHs 2, 3, 6.2, 7, 8, 11, and 12; acetate for pHs 4 and 5; and borate for pHs 9 and 10) were prepared and mixed with CR, according to the amounts described in the experimental part. As shown in Figure S1, only pH 12 resulted in a noticeable and stable color change, going from yellow (blank solution) to deep brown, after the addition of PAR to CR. This effect occurs because, at pH 12, the phenolic group of PAR is deprotonated and assumes the form of a phenolate ion,^37^ which is more reactive and interacts more easily with the CR, forming a stable chromogenic compound responsible for the observed color change. Thus, pH 12 was established as the ideal condition to ensure an intense and stable colorimetric response suitable for all subsequent studies. Scheme S1 illustrates the reaction between PAR and CR, 4-AAP in the presence of ferricyanide, resulting in the formation of a chromogenic product, probably a quinonimine.

### Analytical Parameters

To construct the concentration levels for colorimetric tests, ten different concentrations of PAR were prepared (4.5, 10, 25, 50, 150, 250, 500, 750, 1000, and 2000 mg L^–1^), covering therapeutic (<150 mg L^–1^) and toxic (>150 mg L^–1^) ranges of the serum PAR concentration according to the Rumack–Matthew nomogram, for 4 h after ingestion. These standards together with the CR resulted in noticeable color changes, as illustrated in Figure 1, ranging from light yellow at the lowest concentrations to dark brown at the highest concentrations.

**Figure 1:**
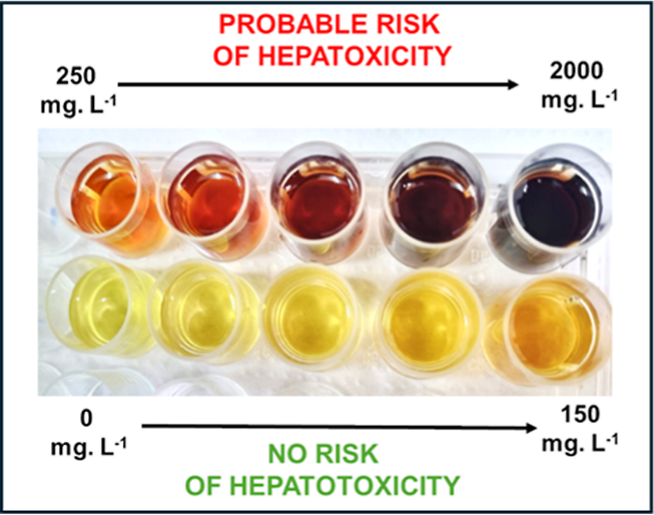
Colors observed for the blank solution and PAR at different concentration levels.

As can be seen in Figure 1, the color change becomes distinctly visible at a concentration of 150 mg. L^–1^. Studies indicate that up to 8 h after PAR ingestion are critical for the best efficacy of the antidote, being the period in which the administration of acetylcysteine has the greatest therapeutic potential.^12^ Considering that within the first 4 h after the PAR ingestion, the lower limit of toxicity is 150 mg L^–1^ ^9^, the proposed colorimetric test is able to detect an intoxication, since the color change becomes evident for this value (Figure 1). This means that up to 4 h postinjection of PAR, it is possible to visually distinguish between the therapeutic range (which remains light yellow) and the toxic range (which begins to acquire orange tones, turning brown at higher concentrations), as shown in Figure 1. However, between 4 and 8 h postinjection of PAR, the toxicity threshold drops to 75 mg L^–1^ ^9^, in which the color change in the colorimetric test becomes imperceptible, and visual interpretation alone may result in incorrect conclusions.

### Electrochemical Method

After the optimal conditions for the colorimetric reaction were established, electrochemical parameters were subsequently optimized to enable sensitive and selective detection of the chromogenic product (PROD).

### Electrochemical Behavior of the Product in the Colorimetric Reaction

With the ideal pH for the colorimetric reaction selected, the electrochemical behavior of PAR was investigated by cyclic voltammetry (CV), before and after the colorimetric reaction in a 0.1 mol L^–1^ phosphate buffer (PB) at pH 12, using SPE-Gr (Figure 2).

**Figure 2:**
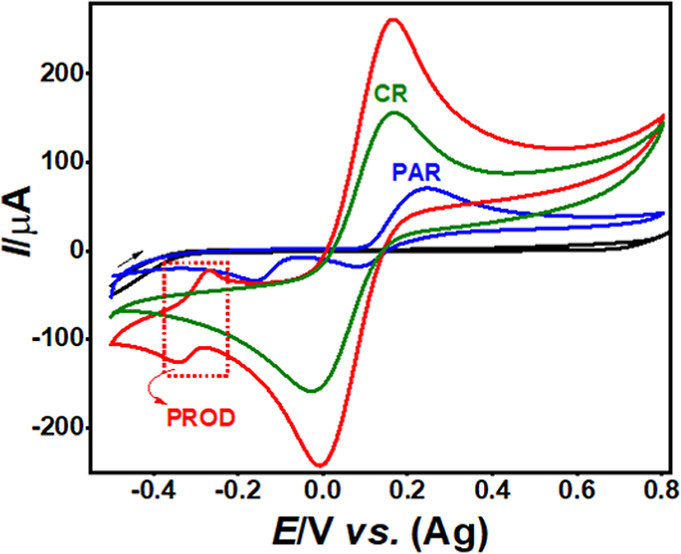
CVs in 0.1 mol L^–1^ PB solution pH 12.0 on SPE-Gr before (black line) and after the addition of 1000 mg L^–1^ PAR (blue-line), CR (green-line), and after the colorimetric reaction between PAR and CR (red-line). Scan rate 50 mV s^–1^.

In a basic medium (PB, pH 12), PAR shows a redox couple (Ox_1_/Red_1_) at around +0.25/+0.02 V (vs Ag), which is assigned to the two-electron oxidation of PAR to the quinone-imine species *N*-acetyl-*p*-benzoquinone imine (NAPQI) and its subsequent reduction back to PAR.^23^ The colorimetric reagent (CR) exhibits a well-defined redox couple at +0.17/-0.01 V (vs. Ag), which is attributed to the Fe (CN)_6_^3–^/Fe (CN)_6_^4–^ redox system. After the chemical reaction between PAR and the CR, a new chromogenic product (PROD) is formed through two sequential steps (CR_1_ and CR_2_), which displays a distinct and reversible redox process at −0.33/-0.27 V (vs. Ag). In the first step (CR_1_), the chromogenic compound is likely formed via the oxidation of the phenolic ring of PAR by K_3_ [Fe (CN)_6_] in a basic medium, accompanied by the loss of the acetamide moiety. This process is followed by the addition of 4-AAP (CR_2_), resulting in the formation of a quinoneimine derivative (PROD). This chemical reaction between PAR and the CR follows a previously reported reaction mechanism^33^ in which the same reagents were employed for the detection of phenylethylamine derivatives (NBOHs). Similar to PAR, these compounds possess a phenolic moiety in their molecular structures, which undergoes an analogous reaction pathway, as confirmed by LC–MS analysis,^33^ leading to the formation of a chromogenic product comparable to that described in the present study. Furthermore, the electrochemical behavior of the PROD shown in Figure 2 is consistent with a quinoneimine-type structure, as proposed in Scheme S1, and it provides a selective analytical signal for PAR determination after the colorimetric reaction. Figure 2 also shows that the redox processes of PAR and CR occur at similar potentials, leading to overlap when both species are present in the same solution (red line). However, the chromogenic product (PROD) formed by the colorimetric reaction presents a distinct and well-defined electrochemical response. The process starts with the reduction of PROD at around −0.33 V (vs. Ag), followed by its oxidation at approximately −0.27 V (vs. Ag), enabling a clear identification of the product signal.

The mass-transport control of the PROD redox reaction at the SPE-Gr surface was evaluated by CV at different scan rates (*v*) in a 0.1 mol L^–1^ PB solution, pH 12.0 (Figure S2). The anodic peak current (*I*_pa_) of PROD was proportional to the scan rate (*R* ^2^ = 0.995, Figure S2B) and to the square root of the scan rate (*R* ^2^ = 0.982, Figure S2C). Furthermore, the logarithmic plot of *I*_pa_ vs *v* revealed a linear relationship.

1 $$y=-3.65\left(\pm 0.02\right)+0.71\left(\pm 0.01\right)x,R^{2}=0.998$$

As the slope value was 0.71, these results indicate that the process is controlled partly by diffusion and partly by adsorption.^39^ Thereby, an AdSV technique associated with DPV and SWV can be used to improve the sensitivity of the proposed method.

Following the identification of the PROD redox peak and given the partial adsorption-controlled behavior observed in the scan-rate study, SWAdSV was selected as the analytical technique and its operational parameters were systematically optimized. The SWAdSV technique was chosen for detecting PROD (after reacting PAR and CR), since SWV offers a higher sensitivity when the electroactive molecules present redox processes. The reaction time was tested from 1 to 5 min, showing maximal PROD formation after 3 min (Figure S3), and this value was therefore adopted for all electrochemical assays. Subsequently, SWAdSV parameters (pulse amplitude, step potential, and frequency) were systematically varied to enhance the signal intensity and peak resolution. An amplitude of 100 mV, a 5 mV step potential, and a frequency of 60 Hz provided the best analytical response. Finally, an accumulation time of 1 min yielded the highest and most reproducible peak currents (Figure S4), consistent with the partially adsorption-controlled behavior of PROD on the SPE-Gr surface. Therefore, the total time for detecting PAR using the electrochemical method is 4 min, a short time crucial for emergency use. The electrochemical profiles of PAR, CR, and PROD at SPE-Gr under these optimized conditions with SWAdSV are shown in Figure 3.

**Figure 3:**
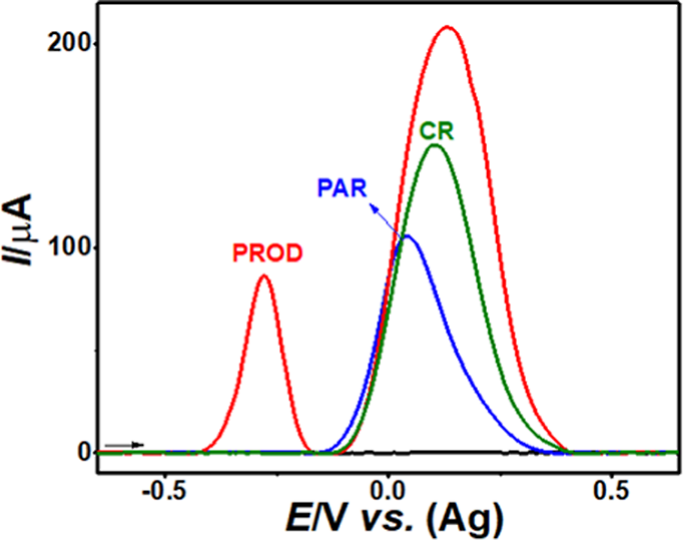
SWAdSVs in 0.1 mol L^–1^ PB solution pH 12.0 (black line) before and after the addition of 150 mg L^–1^ PAR (blue-line), CRs (green-line), and PROD (red-line). Experimental conditions: reaction time: 3 min, amplitude of 100 mV, step potential of 5 mV, frequency of 60 Hz, and accumulation time of 1 min.

Figure 3 shows clearly that the SWAdSV with a SPE-Gr exhibits a separate electrochemical signal for PROD detection. This well-defined and distinct signal allows the monitoring of PAR concentrations in serum samples. The stability of the electrochemical responses was assessed through repeatability studies (Figure 4), where the peak current (*I*p) and peak potential (*Ep*) of the PROD peak were evaluated, using the same electrodes (*n* = 3) and different electrodes (*n* = 3).

**Figure 4:**
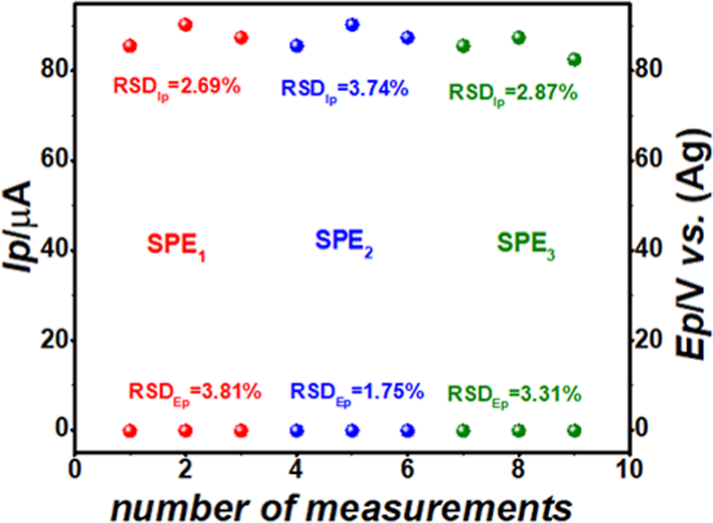
*I*p and *Ep* vs. the number of measurements performed on the same and three different SPE-Gr of the PROD redox process for 150 mg L^–1^ PAR in 0.1 mol L^–1^ PB at pH 12.0 using SWAdSV. Insets are the RSDs (*n* = 3) of *I*p and *Ep* for each SPE. Experimental: 3 min reaction time, 100 mV amplitude, 5 mV step potential, 60 Hz frequency, and 1 min accumulation time.

As shown in Figure 4, the electrochemical PROD responses by SWAdSV using either the same or different SPE-Gr demonstrated low relative standard deviations (RSDs), with an overall RSD for an *I*p of 1.48% (*n* = 3) and an overall RSD for an *E*p of 0.87% (*n* = 3). These results suggest that the combination of a SPE-Gr with the SWAdSV technique offers a stable and reliable method for the detection and quantification of PAR in serum samples using the *I*p and *E*p from the PROD peak, respectively.

The electroanalytical calibration curve to quantify PAR was evaluated between 4.5 and 1000 mg L^–1^, ensuring a broad range of concentrations that span from therapeutic to toxic levels, thereby optimizing the method for practical applications. The concentration range investigated was identical with the one used for the colorimetric method.

As it can be seen in Figure 5, while the colorimetric test presented only a barely visually detectable color change until 150 mg L^–1^ (Figure 5A), the electrochemical technique was able to quantify PAR from 4.5 mg L^–1^ onward, demonstrating a higher sensitivity (Figure 5B). The electrochemical analysis presented a linear range from 4.5 to 1000 mg L^–1^ for the PROD monitoring, with the regression equation

2 $$Ipa=5.0\left(\pm 0.4\right)+0.536\left(\pm 0.009\right)\left[PAR\right],R^{2}=0.996$$

**Figure 5:**
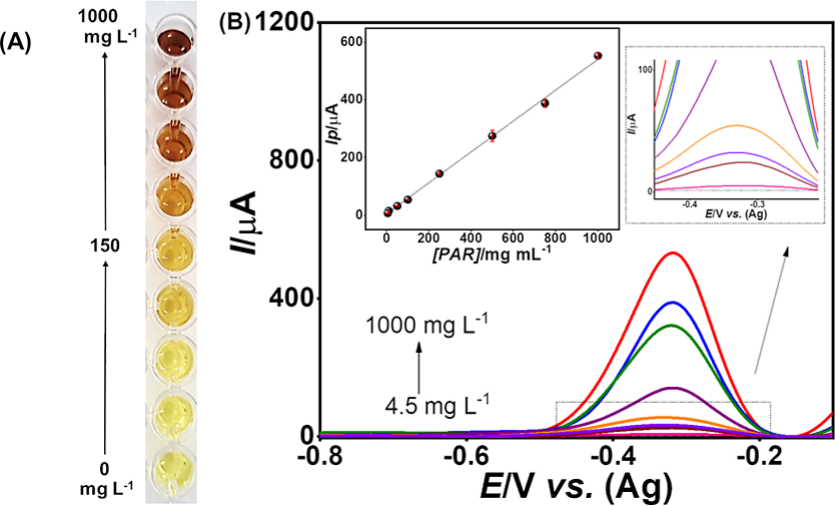
(A) Results of the colorimetric test for PAR solutions in concentrations from 4.5 to 1000 mg L^–1^ and (B) SWAdSV voltammograms of these solutions. Insets are the linear regression of *I*p vs *[PAR]* and the zoom-in view of the lowest measurable signals. Experimental conditions: colorimetric reaction time of 3 min, 100 mV amplitude, 5 mV step potential, 60 Hz frequency, and 1 min accumulation time.

The LOD and LOQ were calculated with 1.2 mg L^–1^ and 4.0 mg L^–1^, respectively. Although the literature describes electrochemical methods with lower LODs,^11^ the clinical relevance of this work lies in its practical applicability for the diagnosis and monitoring of acute PAR poisoning. The proposed method effectively covers the entire spectrum of concentrations evaluated in the clinical protocol for investigating this condition (4.5 to 150 mg L^–1^),^9^ unlike most methods reported in the literature, whose linear working ranges are often narrow and inadequate for the wide variation in PAR concentrations found in biological fluids after poisoning.^11^ As can be seen in the zoomed-in image of Figure 5, the lowest measurable signal (4.5 mg L^–1^) obtained by the proposed method for PAR detection is sufficiently low for an accurate quantification at concentrations of toxicological interest. It is important to emphasize that these low LODs and LOQs previously reported for PAR determination are based on the use of modified electrodes.^11^ In addition, these reported values are theoretical and often deviate substantially from the lowest concentration levels that can be reliably measured for PAR determination by respective working linear ranges.^11^ On the other hand, our combined method uses a portable and unmodified electrode, which can be used as a simple point-of-care test for selective detection and quantification of PAR in toxicological analysis, representing an advance in relation to the more complex approaches previously reported.

To further expand the method’s versatility, an additional analytical curve was constructed for the PAR detection in the absence of CR (Figure S5). This provides a complementary monitoring option. As the electrochemical processes of PAR and CR at a SPE-Gr overlap, this additional curve allows a complementary analysis that can be performed before the colorimetric test. This approach can also offer a triple identification of PAR, using one information by the colorimetric test (color change) and further two responses by the electrochemical detection of PAR and PROD, obtained using SWAdSV before and after the addition of CR, respectively. The linear range obtained only for the PAR detection was from 10 to 1000 mg L^–1^, with the regression equation

3 $$Ip=-1.6\left(\pm 0.2\right)+0.386\left(\pm 0.007\right)\left[PAR\right],R^{2}=0.998$$

The LOD and LOQ were calculated with 3.0 mg L^–1^ and 9.9 mg L^–1^, respectively, ensuring that the method is sensitive enough for a detection at clinically relevant levels. Thereby, the combined colorimetric–electrochemical detection provides a selective identification and quantification of PAR, with double confirmation of the presence of this drug, with (1) the initial color change identifies the presence of PAR at toxic levels, typically observed within 4 h after ingestion; and (2) the redox process between PAR and CR forming PROD, which subsequently allows the electrochemical identification and monitoring of PAR concentrations in serum samples.

### Selectivity toward Clinically Relevant Interferents

For the interference study, potential interferents were selected based on their clinical relevance in acute PAR intoxication and their reported electrochemical activity in serum.^38^ The electrochemical behavior of each compound was first evaluated individually (i.e., in the absence of PAR) after application of the colorimetric protocol, as illustrated in Figure S6A. Each interference was tested at concentrations close to the reference serum levels to better reflect real clinical conditions. Ascorbic acid (AA) was tested at 20 mg L^–1^, slightly above the reference range of 4–20 mg L^–1^,^40^ while uric acid (UA) was tested at 70 mg L^–1^, corresponding to the upper normal limit for men (70 mg L^–1^) and above the reference range for women (60 mg L^–1^).^41^ Caffeine (CAF) was tested at 20 mg L^–1^, the upper limit of the serum range (8–20 mg L^–1^).^42^ Glucose (GLI) was tested at 1100 mg L^–1^, slightly above the reference range of 700–1050 mg L^–1^.^43^ In addition, potential exogenous interferents that may co-occur with paracetamol in acute poisonings—aspirin (SA), diazepam (DIAZ), and codeine (COD)^38^—were investigated at 200 mg L^–1^.

Figure S6A shows that under the optimized conditions of the proposed method, AA, AU, CAF, GLI, SA, DIAZ, and COD did not produce any visible color change, indicating that they do not react with the colorimetric reagent (CR). Consistently, the SWAdSV voltammograms (Figure S6A) show that these compounds do not exhibit electrochemical processes near the PROD signal at the SPE-Gr electrode, further supporting the absence of reactions with CR. Figure S6B further demonstrates that when PAR is mixed with each interferent, the colorimetric reaction between PAR and CR is preserved, as evidenced by the visible color change and by the appearance of the characteristic PROD electrochemical signal in the corresponding SWAdSV voltammograms. Therefore, none of these compounds interfered with the identification of PAR using the combined colorimetric-electrochemical approach.

As summarized in Figure S6C, the peak current (*I*p) values associated with the PROD redox process at the SPE-Gr electrode, recorded in the absence and presence of each interferent, yielded recoveries close to 100%, indicating no significant interference in the quantification of PAR. Overall, the proposed method enables accurate identification and quantification of PAR even in the presence of common potential interferents at concentrations within clinical reference ranges. This selectivity addresses a recurring limitation in earlier electrochemical methods for PAR determination, where the influence of interferents at realistically relevant concentrations in biological matrices is often not evaluated.^11^

This panel was designed to reflect the most clinically relevant species for acute intoxication and point-of-care screening. Depending on the intended clinical setting, additional comedications (including selected antibiotics) may also be evaluated in future works.

From a practical standpoint, the proposed colorimetric-electrochemical platform aligns well with routine clinical workflows for managing acute paracetamol intoxication. In an emergency, the rapid visual colorimetric response can assist early triage by indicating potentially toxic concentrations within the first hours after ingestion. The subsequent electrochemical measurement provides quantitative confirmation at the point of care, requiring neither specialized personnel nor laboratory instrumentation. Compared with commonly used rapid assays such as ELISA—whose operation typically requires 20–40 min, dedicated reagents, and trained staff—or spectrophotometric tests, which depend on centralized laboratory resources, the total analysis time of approximately 4 min represents a significant improvement. This operational simplicity, combined with compatibility with commercial disposable electrodes, underscores the feasibility of implementing the method as a practical bedside or near-patient tool in emergency toxicology settings.

### Determination of PAR in an Authentic Serum Sample

For an application in human serum samples, analyses were performed using the colorimetric and electrochemical methods, with the aim of verifying the viability of the method under clinical conditions. A human serum sample with the suspected PAR intoxication was analyzed. This sample was also spiked with 10 mg L^–1^ of PAR to perform addition-recovery studies using the proposed method. Due to the observed matrix effect, standard addition curves were recorded for the PAR quantification in the serum sample using the proposed method.

The human serum sample was initially subjected to an electrochemical analysis before the colorimetric reaction for a PAR quantification via its electrochemical signal at a SPE-Gr. Addition-recovery studies were performed with the addition of PAR in this serum sample at concentrations of 10, 150, and 750 mg L^–1^, resulting in a recovery of 114.7 (±8.6) %, 96.4 (±2.6) %, and 106.0 (±5.6) %, respectively (Figure S7). These results indicate that the electrochemical methodology is suitable for PAR quantification in human serum samples, prior to the colorimetric reaction. In a second step, the colorimetric test was performed on the same human serum sample with the addition of PAR at 10, 50, 150, and 750 mg L^–1^. Due to the low concentration of 10 mg L^–1^ of PAR, there was no noticeable color change, as shown in the inset in Figure S8A, since this concentration is below the visual detection limit by the proposed method. However, as can be seen in Figure S8A, the human serum yielded a clear and stable color change beyond 150 mg L^–1^. Thus, the visual color test can identify the risk of intoxication in analysis situations performed up to 4 h after PAR ingestion, as indicated by the Rumack–Matthew nomogram.^9^

The confirmation of the toxicity of this serum sample was conducted after colorimetric reaction by the electrochemical method. Analyses by SWAdSV with a SPE-Gr were performed (Figure S8B) with the addition of 10, 150, and 750 mg L^–1^ PAR in the same serum sample, obtaining a recovery between 80 and 120%. Therefore, the electrochemical method after the colorimetric reaction demonstrated the ability to quantify PAR at concentrations aligned with the toxicity range described by the Rumack–Matthew nomogram,^9^ covering up to 19 h after ingestion. This approach is especially relevant in clinical scenarios where the serum PAR concentration needs to be monitored for longer periods after ingestion, overcoming the limitations of the initial colorimetric test. The application in the human serum is presented as a proof of concept to demonstrate the feasibility in a real matrix. Further validation using a larger set of clinical samples and different electrode lots would be required to fully establish the robustness for routine implementation.

Given the simplicity of the workflow and the use of commercially available SPEs, the method is readily adaptable to portable or fully integrated point-of-care devices. The colorimetric and electrochemical steps could be incorporated into a single disposable cartridge or a compact hand-held reader, enabling bedside testing without laboratory infrastructure. Such integration would further reduce turnaround times and support rapid toxicological decision-making in emergency settings.

Beyond its analytical performance, the relevance of the proposed method lies in its alignment with real clinical needs for managing acute PAR intoxication. The integrated colorimetric–electrochemical approach was deliberately designed to operate within the serum concentration range used in clinical practice to guide diagnosis and treatment according to the Rumack–Matthew nomogram. Thus, unlike many reported sensors that emphasize ultratrace detection outside clinically meaningful ranges, this strategy directly targets the concentration window required for emergency decision-making, while providing dual confirmation of PAR through the formation and electrochemical interrogation of a single chromogenic/electroactive reaction product.

In addition, the method offers practical advantages, including low cost, rapid response, and operational simplicity. By a combination of a classical colorimetric reagent with commercially available disposable SPEs-Gr without surface modification, the proposed approach avoids nanomaterials, complex fabrication steps, and costly instrumentation. These attributes make it a more accessible alternative to hospital-based techniques that often rely on centralized analyzers or limited-access immunoassays, particularly in resource-limited healthcare settings. The use of mass-producible disposable electrodes and straightforward reagent preparation also supports scalability and potential implementation in a kit format, facilitating translation into point-of-care applications. A summary of key performance indicators and the quantified gains achieved by the proposed method is provided in Table S1 (Supporting Information).

## Conclusion

This study addresses the need for rapid, reliable, and accessible tools for assessing acute PAR intoxication, particularly in cases where conventional laboratory methods are constrained by cost, infrastructure, or turnaround time. By operating within the clinically relevant serum concentration range used in the Rumack–Matthew nomogram, the proposed approach bridges an important gap between sensor development and actionable clinical decision-making. The key advance is the integration of a simple colorimetric assay with electrochemical detection on unmodified disposable SPEs, enabling the dual confirmation of PAR through a single reaction product. The method combines low cost, fast analysis, operational simplicity, and selectivity under clinically relevant interference conditions, supporting scalability and potential translation to point-of-care testing for PAR intoxication management.

## Supplementary Material



## Notes

Proposed redox mechanism for PAR; behavior of the colorimetric reagent as a function of pH; effect of scan rate on the electrochemical response of PAR; influence of colorimetric reaction and accumulation times on the PROD peak current; PAR calibration curve; colorimetric results, SWAdSV voltammograms, and recovery data for PAR, interferents, simulated PAR-interferent mixtures, and real-sample scenarios; and key performance indicators and quantified gains (PDF)
