# Supplementary material for: Rapid Point-of-Care Assessment of Paracetamol Intoxication via an Integrated Electrochemical–Colorimetric Method
Source: ACS Omega. 2026 Feb 24;11(9):15094–102. doi: 10.1021/acsomega.5c12147 (PMC12980178; doi:10.1021/acsomega.5c12147)
Supplement: Supplementary file 1 [file ao5c12147_si_001.pdf]

SUPPLEMENTARY INFORMATION

Rapid Point-of-Care Assessment of Paracetamol Intoxication via an Integrated Electrochemical–Colorimetric Method

*Anne A. Macedo[a], Dilton M. Pimentel[b], Karla A. O. Souza[e,f], José L. Costa[f,g], Cláudia M. Rocha,[c] Clésia C. Nascentes[c], Ângelo de Fátima[c], Luciano C. Arantes[d], Fateme Ebrahimi[h], Bertold Rasche[h], Wallans T. P. dos Santos[a,h]**

[a]*Departments of Chemistry and Pharmacy, Universidade Federal dos Vales do Jequitinhonha e Mucuri (UFVJM), 39100000, Diamantina-MG, Brazil*

[b]*Laboratório Integrado de Pesquisas do Vale do Jequitinhonha, PRPPG-UFVJM, 39100000 Diamantina-MG, Brazil.*

[c]*Department of Chemistry, Instituto de Ciências Exatas, Universidade Federal de Minas Gerais (UFMG), 31270-901, Belo Horizonte-MG, Brazil.*

[d]*Instituto Nacional de Ciência e Tecnologia sobre Substâncias Psicoativas (INCT-SP), UFMG, 31270-901, Belo Horizonte-MG, Brazil.*

[e]*Faculdade de Ciências Médicas, Universidade Estadual de Campinas (UNICAMP),13083-859 Campinas-SP, Brazil*

[f]*Centro de Informação e Assistência Toxicológica de Campinas (CIATox-Campinas), UNICAMP,13083-859 Campinas-SP, Brazil*

[g]*Faculdade de Ciências Farmacêuticas, UNICAMP,13083-859 Campinas-SP, Brazil*

[h]*Institute of Inorganic Chemistry, University of Stuttgart, 70569 Stuttgart, Germany*

*Corresponding author:

Email: wallanst@ufvjm.edu.br

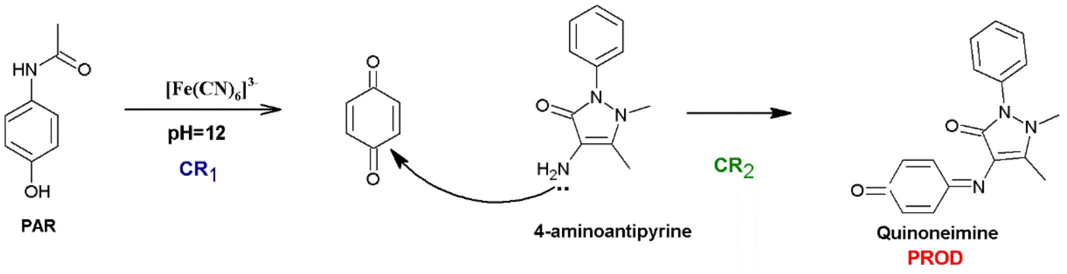

Scheme S1: Proposed reaction between CR and PAR in a phosphate buffer at *pH* 12.

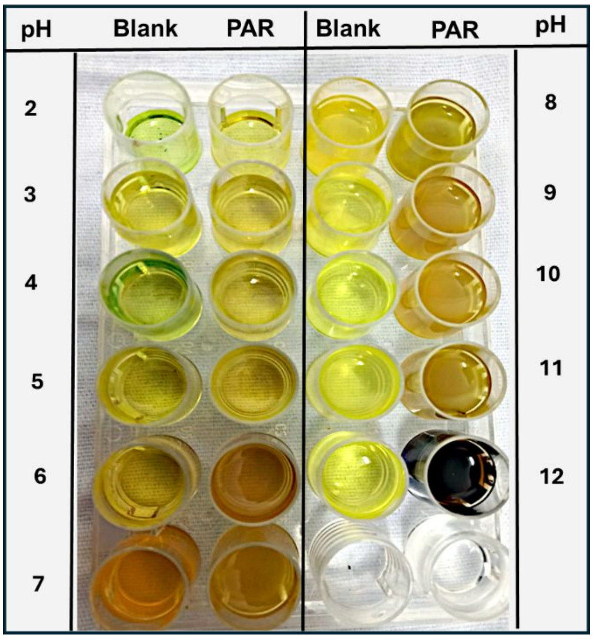

Figure S1. Color variation in the colorimetric test for PAR 2000 mg L^{-1} at different *pH* values (2 to 12). The first column shows the blank solutions in the presence of the colorimetric reagent (CR), while the second column shows the PAR solutions in the presence of CR.

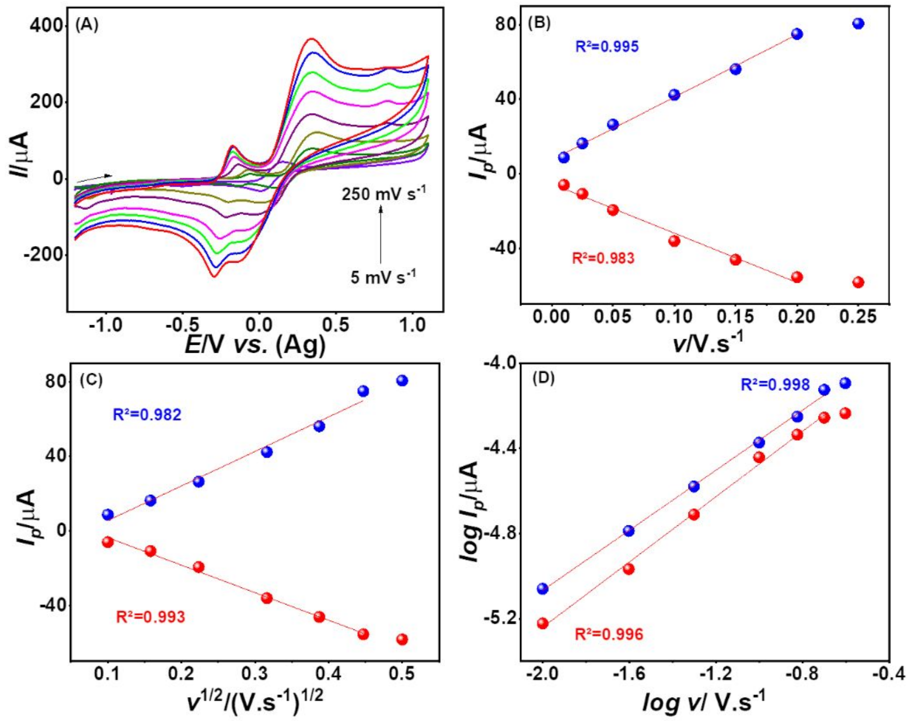

Figure S2. Influence of the scan rate on the electrochemical response. **(A)** CVs of 1000 mg L^{-1} PAR+CR in a 0.1 mol L^{-1} phosphate buffer solution at *pH* 12.0 at a SPE-Gr. Potential scans started at −1.25 V in the anodic direction, see arrow. Scan rates (v) ranged from 5 mV s^{-1} to 250 mV s^{-1}. Linear regressions of **(B)** *Ip* vs. *v*, **(C)** *Ip* vs. $v^{1/2}$, and **(D)** log (*Ip*) vs. log(*v*).

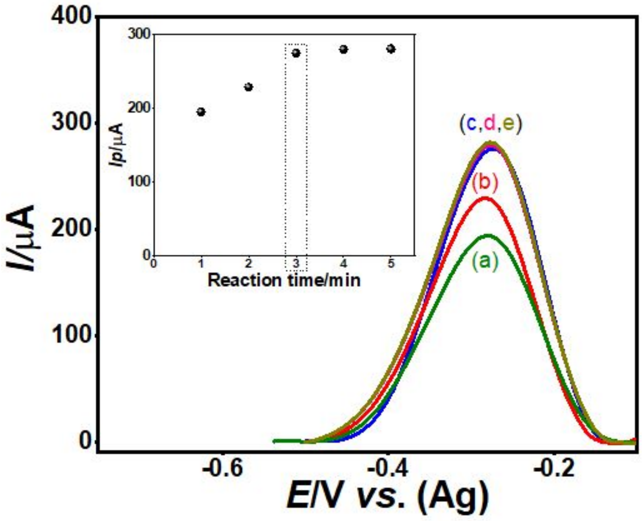

Figure S3. SWAdSVs of PROD 1000 mg L^{-1}, in a phosphate buffer medium, *pH* 12, after different colorimetric reaction times: (a) 1 min, (b) 2 min, (c) 3 min, (d) 4 min and (e) 5 min. The insert is *Ip* vs. *Reaction time.* Experimental conditions: amplitude of 100 mV, step potential of 5 mV, frequency of 60 Hz.

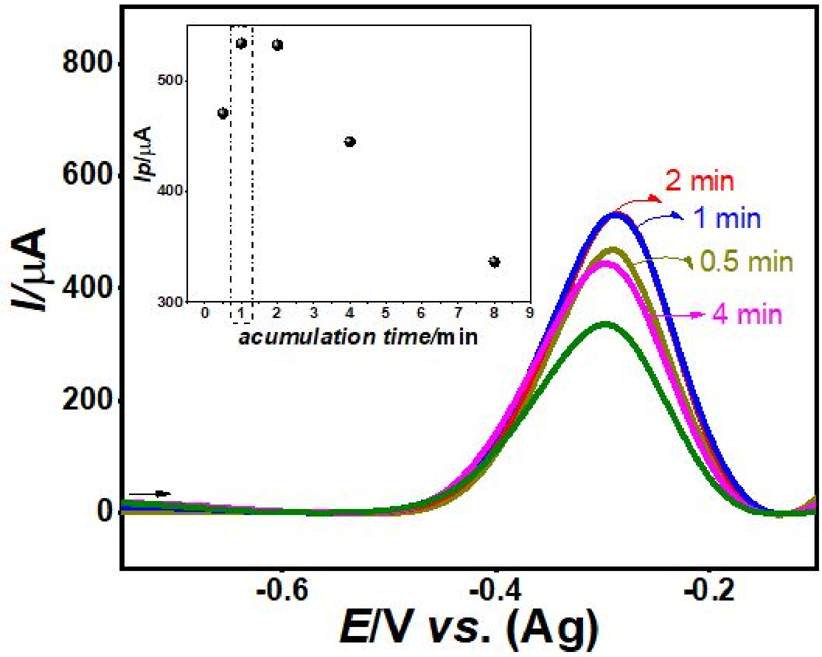

Figure S4. SWAdSV voltammograms of PROD at a concentration of 1000 mg L^{-1} in 0.1 mol L^{-1} phosphate buffer solution at *pH* 12.0 at a SPE-Gr. The accumulation time varied from 0.5 to 2.0 min. The inset illustrates the *Ip* vs. *accumulation time* relationship. Experimental conditions: reaction time: 3 min, amplitude of 100 mV, step potential of 5 mV, frequency of 60 Hz.

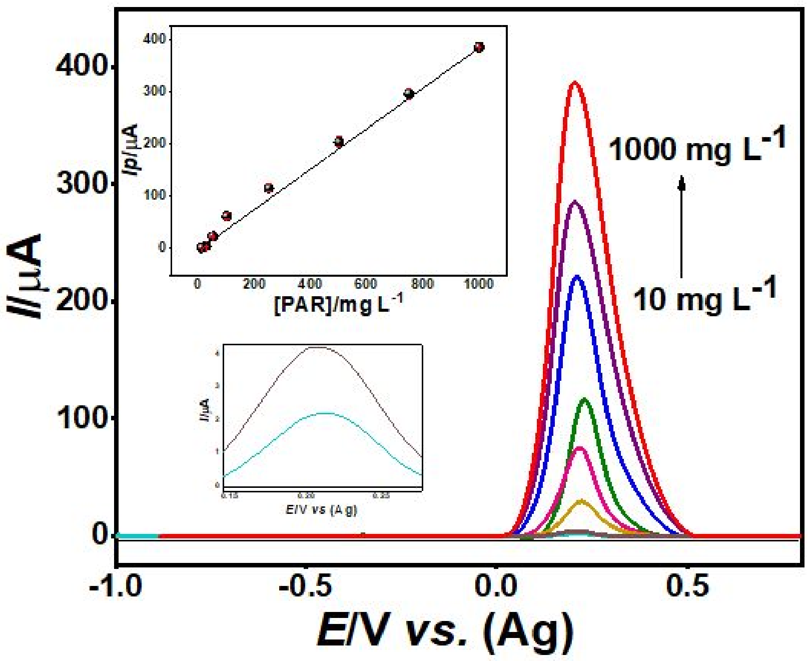

Figure S5. SWAdSV voltammograms of PAR solutions from 10 to 1000 mg L^{-1}. Inserted linear regressions *Ip* vs. *[PAR]* and a magnified view of the smallest measurable signals. Experimental conditions: reaction time: 3 min, amplitude of 100 mV, step potential of 5 mV, frequency of 60 Hz, and accumulation time of 1 min.

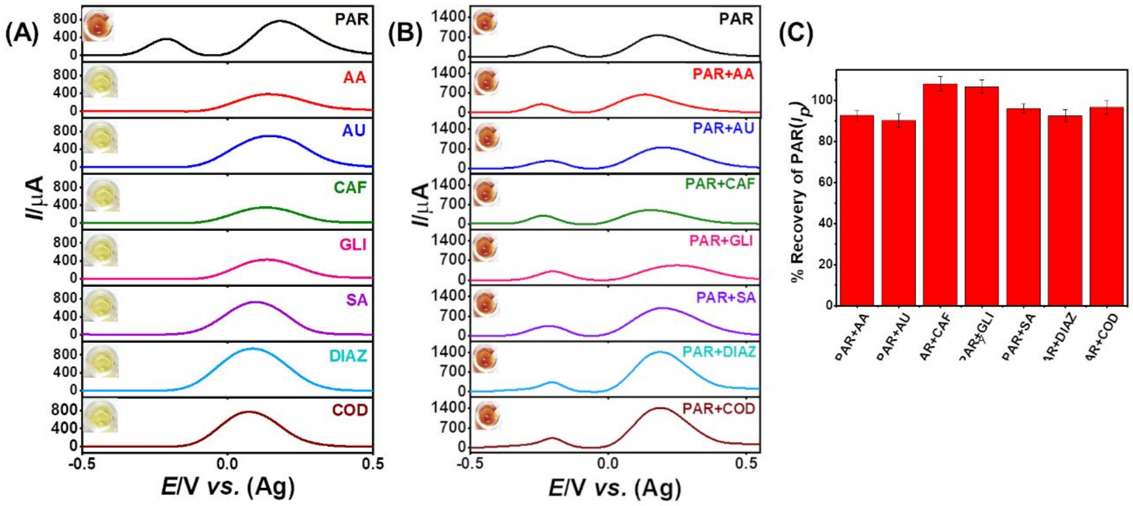

Figure S6. (A) Representative SWAdSV voltammograms recorded for PAR (1000 mg L^{-1}; black line) and for each investigated interferent measured individually after applying the colorimetric procedure, together with the corresponding visual colorimetric tests (insets). **(B)** Representative SWAdSV voltammograms of PAR in the presence of each investigated interferent under the same experimental conditions, also accompanied by the corresponding visual scolorimetric tests (insets). **(C)** Recovery of the peak current (*Ip*) associated with the PROD redox process in the presence of each investigated interferent. Legend abbreviations: paracetamol (PAR); ascorbic acid (AA); uric acid (AU); caffeine (CAF); glucose (GLI); salicylic acid (SA); diazepam (DIA); codeine (COD).

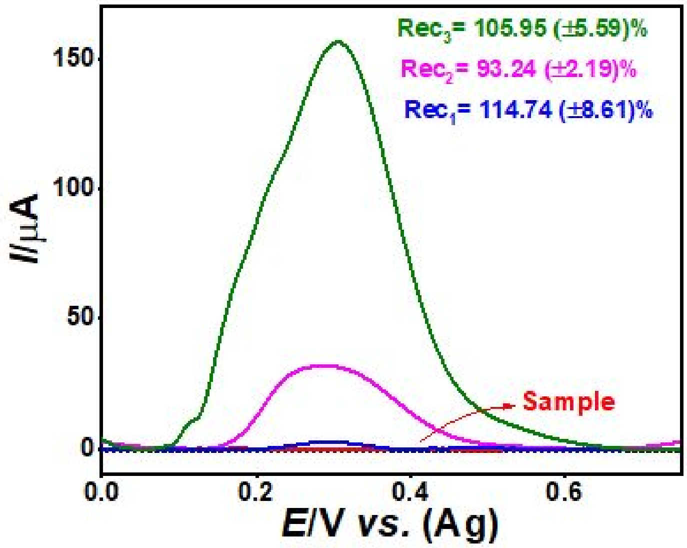

Figure S7. Voltammetric profile of authentic human serum sample with additions of 10 (blue line), 150 (pink line) and 750 (green line) mg L^{-1} of PAR standard. Insets are the recovery values obtained for each addition. Experimental conditions: reaction time: 3 min, amplitude of 100 mV, step potential of 5 mV, frequency of 60 Hz, and accumulation time of 1 min.

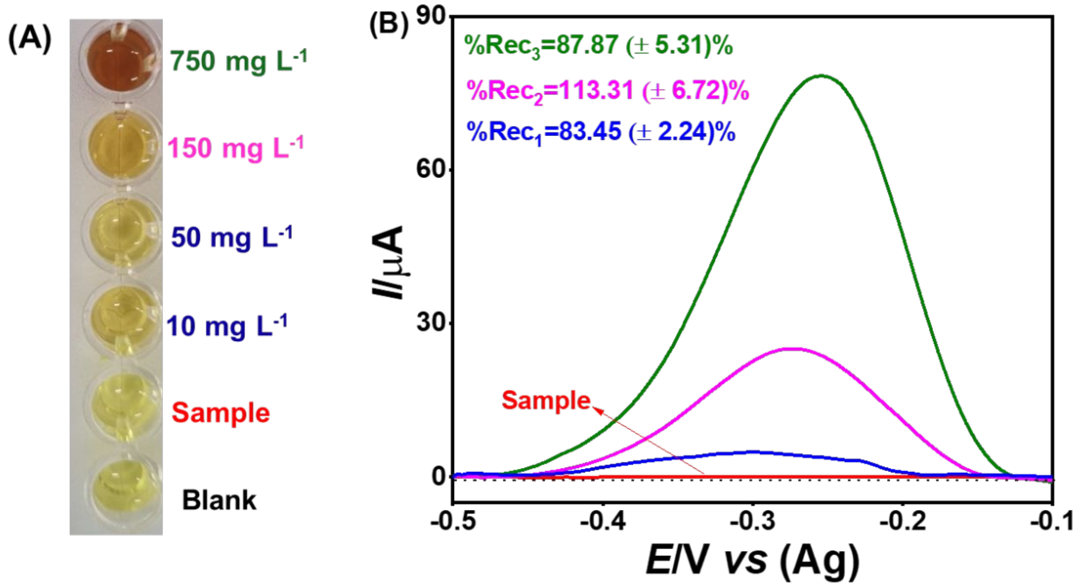

Figure S8. (A) Result of the colorimetric test for: colorimetric reagents (blank), authentic human serum sample with additions of 10, 50, 150 and 750 mg L^{-1} of PAR. **(B)** Voltammetric profile of authentic human serum sample (red line), with additions of 10 (blue line), 150 (pink line) and 750 mg L^{-1} (green line) of PAR standard.

Table S1. Key performance indicators (KPIs) and quantified gains of the proposed colorimetric–electrochemical method for paracetamol determination.

Parameter	This work	Practical relevance
Detection concept	Colorimetric + electrochemical (dual confirmation)	Improves reliability and reduces false positives
Linear range	4.5-1000 mg L^{-1}	Direct applicability to acute intoxication diagnosis (Rumack–Matthew nomogram)
Response time	< 5 min	Suitable for emergency screening
Electrode	Commercially available, unmodified SPE-Gr	Simple, portable, low-cost, and scalable.
Repeatability	RSD < 4% (n=3)	Adequate stability
Reproducibility	RSD < 2% (n=3)	Adequate stability
Sample matrix	Human serum	Clinically relevant validation
Sample preparation	Simple dilution in the supporting electrolyte	Minimal handling, point-of-care compatible
